# Framework for industry engagement and quality principles for industry-provided medical education in Europe

**DOI:** 10.1080/21614083.2017.1348876

**Published:** 2017-07-31

**Authors:** Tamara Allen, Nina Donde, Eva Hofstädter-Thalmann, Sandra Keijser, Veronique Moy, Jean-Jacques Murama, Thomas Kellner

**Affiliations:** ^a^ Medical Education Department, Eli Lilly and Company Europe, Sunningdale, UK; ^b^ Medical Education Department, Novo Nordisk, Gatwick, UK; ^c^ Medical Education Department, Janssen the Pharmaceutical Company of J&J Europe, Vienna, Austria; ^d^ Medical Education Department, Genzyme Europe BV, Naarden, The Netherlands; ^e^ Medical Education Department, Merck KGaA, Darmstadt, Germany; ^f^ Medical Education Department, Eli Lilly and Company Europe, Geneva, Switzerland; ^g^ Medical Education Department, UCB Biosciences, Monheim, Germany

**Keywords:** Lifelong learning, medical education, continuing professional development, knowledge transfer, pharmaceutical industry engagement, quality education

## Abstract

Lifelong learning through continuing professional development (CPD) and medical education is critical for healthcare professionals to stay abreast of knowledge and skills and provide an optimal standard of care to patients. In Europe, CPD and medical education are fragmented as there are numerous models, providers and national regulations and a lack of harmonisation of qualitative criteria. There is continued debate on the appropriate role of pharmaceutical companies in the context of medical education. Accrediting bodies such as European Accreditation Council for Continuing Medical Education do not permit active involvement of the pharmaceutical industry due to concerns around conflicts of interest and potential for bias. However, many examples of active collaboration between pharmaceutical companies and medical societies and scientific experts exist, demonstrating high integrity, clear roles and responsibilities, and fair and balanced content. Medical education experts from 16 pharmaceutical companies met to develop a set of quality principles similar to standards that have been established for clinical trials and in alignment with existing principles of accrediting bodies. This paper outlines their proposal for a framework to improve and harmonise medical education quality standards in Europe, and is also an invitation for all stakeholders to join a discussion on this integrative model.

## Introduction

In an increasingly complex healthcare environment, with major scientific and technical innovations, aging populations, increasing spread of chronic disease and major cost constraints, healthcare professionals (HCPs) are required to keep up-to-date with new data and advances in order to ensure optimal patient care [1]. Consequently, patient care needs a multidisciplinary approach, including clinical decision-making, clinical management and patient support [1], which involves an intricate relationship between all healthcare stakeholders and the pharmaceutical industry.

Medical education for practising HCPs in Europe is driven by different models, various providers and national regulations, and hence is very fragmented [2–4]. In the majority of European countries, medical education is provided by universities, physician associations/societies, medical education/communication companies and the pharmaceutical industry [4], all of whom have a shared interest in enabling the delivery of optimal standards of care for patients [5]. Leading European medical societies value strong collaboration between many different parties – including pharmaceutical companies – to ensure that knowledge transfer and continuing medical education (CME) and continuing professional development (CPD) programmes are accessible to HCPs to improve patient care [6]. Within pharmaceutical companies there is in-depth knowledge and expertise in clinical development, disease areas and healthcare systems, and increasingly in educational science [7], and companies are conscious of the importance of providing accurate, fair and objective information about medicinal products so that rational decisions can be made as to their use [8].

However, over the last decade engagement by pharmaceutical companies has been challenged by accrediting bodies, government regulatory agencies, academic bodies and the public in general [9–14]. Examples of inappropriate use of medical education for promotional purposes and inherent bias due to commercial conflicts of interest have overshadowed many other constructive initiatives and collaborations that accentuate the positive contribution of the pharmaceutical industry in the lifelong learning of European HCPs.

Considering the above points, how can the pharmaceutical industry have a more widely accepted and legitimate role in developing and supporting medical education for HCPs? Similar to the standards that have been implemented for clinical trials, this paper is the first attempt by medical educational experts within the pharmaceutical industry to discuss a potential framework for industry engagement in Europe.

## Pharmaceutical industry quality principles

**Figure 1.**
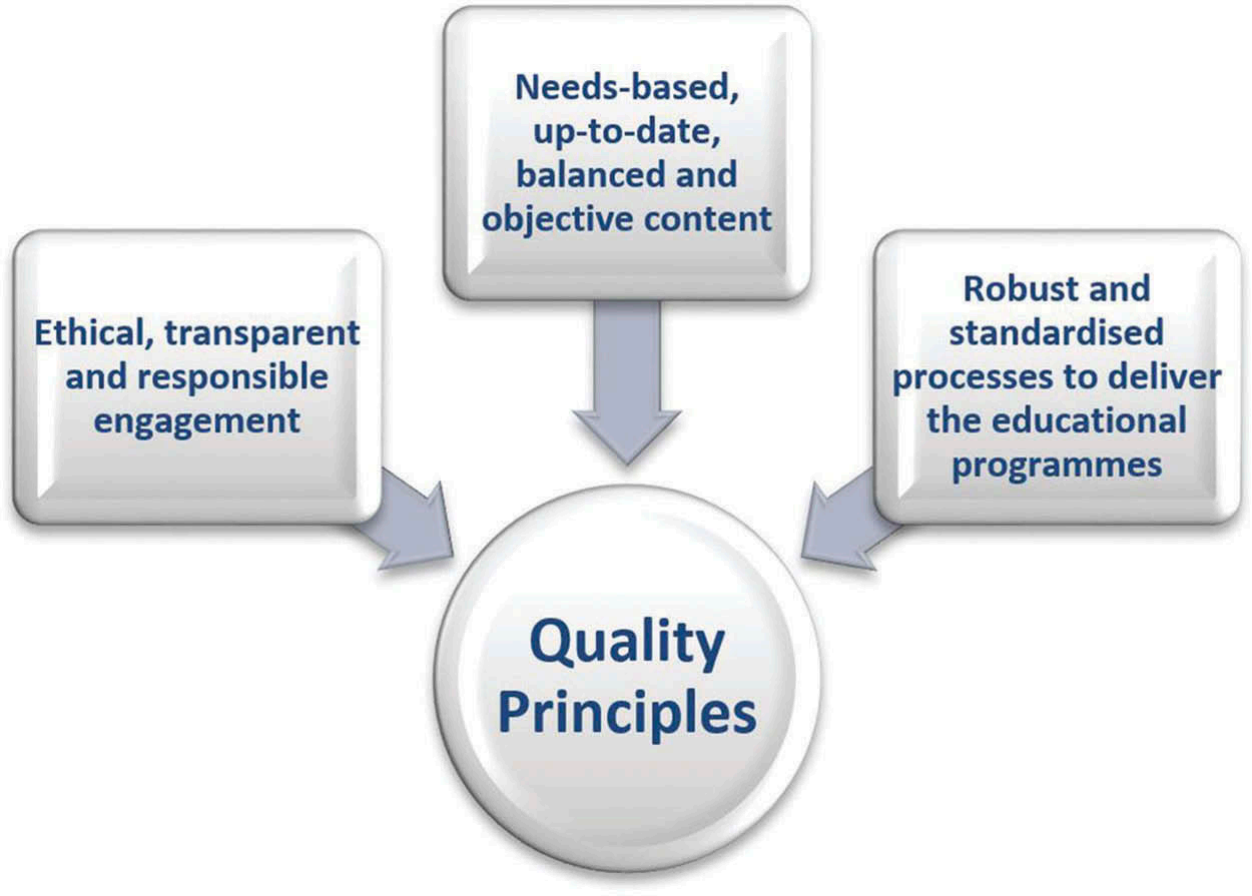
Elements in delivering high-quality medical education.

Driven by the strong interest of 16 pharmaceutical companies to align on a common set of quality principles for medical education provided or supported by the pharmaceutical industry, the authors have consolidated their input into the quality principles outlined herein. In the context of this document, “quality” is defined as the effectiveness of an educational activity or programme in achieving the pre-defined educational objectives. The elements required to deliver high-quality medical education include: ethical, transparent and responsible engagement; needs-based, up-to-date, fair, balanced and objective content; and robust and standardised processes to deliver the educational programmes (Figure 1). These are explained in more detail in Table 1.

**Table 1.** Quality principles for medical education.

Elements	Description
Ethical, transparent and responsible engagement [15,16]	Transparency regarding funding
	Transparency regarding roles and responsibilities
	Disclosure of interests and resolution of potential conflicts
	No disguised promotion
	No attempt to influence expert committee or faculty decisions
	Compliance with EFPIA and local codes of practice, including Disclosure of Transfer of Value codes
	Adherence to data-privacy legislation
	Compliance with research ethics requirements, data-protection legislation and copyright arrangements
	Compliance with anti-bribery and corruption policies
Needs-based, up-to-date, balanced and objective content	Needs-based; i.e. validation by literature, programme scientific committee or a dedicated educational needs assessment
	Provision of scientifically balanced perspectives on the subject matter with involvement of scientific committees when appropriate
	Use of the most appropriate, current and evidence-based content
	Use of adult learning principles [17,18]
	Relevant to learners
	Applicable to clinical practice
Robust and standardised processes to deliver the	Educational needs assessment with the defined target group
educational programmes [19–26]	Application of the principles of instructional design [27]
	Definition of:
	intended programme objectives
	clear and measurable learning objectives
	the target audience and their responsibilities
	Identification of the most effective educational format
	Development of content
	Programme deployment
	Identification and responsible engagement of learners according to strict selection criteria based on educational needs
	Delegation of financial support for travel and housing (direct or indirect sponsorship) must follow EFPIA/local regulations and company compliance codes
	Mechanisms to increase learning impact
	Tools to enhance learners’ active involvement
	Recognition of learning styles
	Expert committee and faculty responsible for planning, developing and reviewing agenda and content based on educational objectives
	Clear and transparent procedures to ensure declaration of interests and resolution of any conflicts prior to the activity
	Outcomes measurement (evaluation of knowledge gain as a minimum requirement) according to the levels determined by the Moore model [19]

For the development of the criteria outlined in Table 1, scientific references, codex and guiding principle publications have been referenced as applicable. EFPIA, European Federation of Pharmaceutical Industries and Associations.

The authors’ proposed pharmaceutical industry quality principles are closely aligned with criteria required by the European Union of Medical Specialists (UEMS)/European Accreditation Council for CME (EACCME) [20], [Table 2]. There are, however, two main differences between the EACCME standards and our proposed quality principles: first, the EACCME criteria exclude any active involvement of the pharmaceutical industry, and second, the quality principles as outlined here have greater emphasis on learning design, transparency and maximising the impact of education.

**Table 2.** Summary of the key EACCME accreditation criteria.

Educational objectives	Needs assessment
	Target audiences
	Expected educational outcomes
	Adult learning principles
Description	Active learning
	Evaluation system per learner
	Ethical, legal, regulatory requirements
	Appointed medical lead
Scientific committee	Appointed
	Declaration of potential conflict of interest
	Resolution of potential conflict of interest
Faculty	Declaration of potential conflict of interest
Programme	Needs to be provided
Funding	Exclusion of pharmaceutical companies as a provider
	Source to be declared
	Content free of influence by sponsor
General	Data-privacy protection
	Compliance with national rules and regulations
Promotional material	Educational material free of promotion
Transfer of value	For all delegates and faculty according to EFPIA Disclosure code is not required

EACCME, European Accreditation Council for Continuing Medical Education; EFPIA, European Federation of Pharmaceutical Industries and Associations. Adapted with permission from [20].

## Scope of engagement

**Figure 2.**
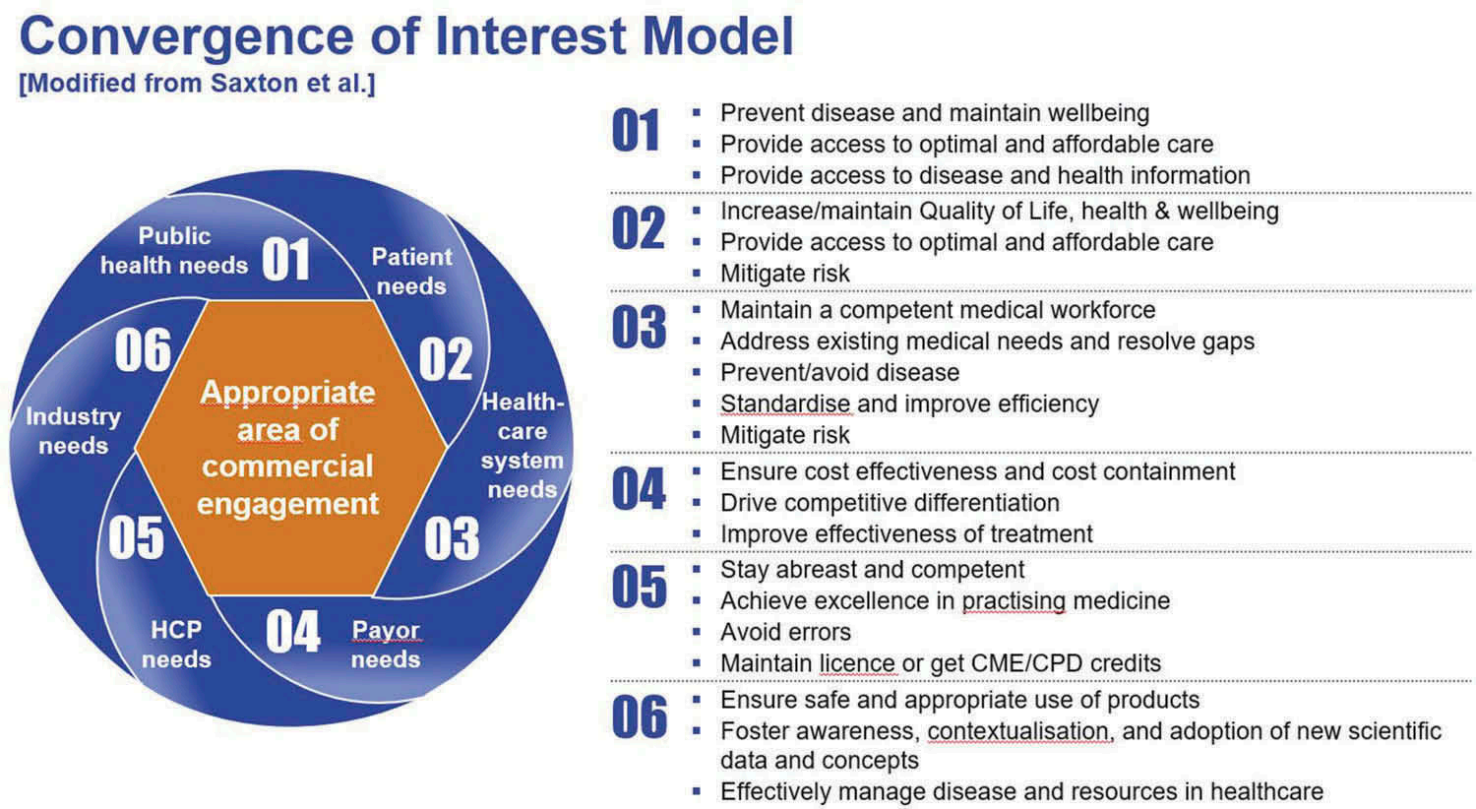
Convergence of interest model: appropriate area of commercial engagement.

It is recognised that in general HCPs, patients and the healthcare system have varying educational needs and goals; however, it is the authors’ experience that there is common consensus that all activities must aim to improve public health, patient outcomes and/or core competencies of HCPs, regardless of the category of industry-provided education. The programmes currently provided/supported by the pharmaceutical industry are most commonly determined by an area of shared interest between learners and providers. This is shown in the “Convergence of Interests Model” (Figure 2) [28].

When considering the current scope of engagement by the pharmaceutical industry in providing educational activities for Europe, the authors have grouped engagement into four categories, which are further explained in detail below:

- Industry-provided product-specific training
- Industry-provided professional development and medical disease education
- Collaborative partnerships
- Support to third-party-provided medical education

### Industry-provided product-specific training (A)

As mentioned above, the pharmaceutical industry is conscious of the importance of providing accurate, fair and objective information about medicinal products so that rational decisions can be made as to their use. It is within this category of educational initiatives that pharmaceutical companies provide training to HCPs specifically related to developed and marketed products. Pharmaceutical companies are the main providers for this type of training. The scope of this engagement follows the product label and respective regulatory requirements. Faculty and content are determined by the company organising the activity, and any product-specific training programmes, materials and content must pass internal review by various relevant stakeholders (e.g., medical, legal, regulatory, compliance) to ensure accurate representation within product licenses.

### Industry-provided professional development and medical disease education (B)

In disease areas where professional competency gaps have been identified or scientific developments warrant further discussion and understanding, the pharmaceutical industry directly provides medical educational CPD programmes for HCPs aligned with the quality principles outlined above. The scientific programme and faculty selection are developed under the guidance of an external steering committee comprised of relevant scientific experts, and overall “ownership” and accountability for these programmes within pharmaceutical companies lies with their medical-scientific departments.

### Collaborative partnerships (C)

The words “collaboration” and “partnership” are often interchangeable when considering aspects of different parties working together (Table 3) [29]. There are many attributes that collaboration and partnership share in common. The collaboration/partnership includes a commitment to a definition of mutual relationships and goals; a jointly developed structure and shared responsibility; mutual authority and accountability for success; and sharing of resources and rewards [30].

**Table 3.** Attributes of partnership and collaboration.

	Partnership	Collaboration
Defining attributes	Trust and confidence in accountability	Trust and respect in collaborators
	Respect for specialist expertise	Joint venture
	Joint working	Teamwork
	Teamwork	Intellectual and cooperative endeavour
	Blurring of professional boundaries	Knowledge and expertise more important than role or title
	Members of partnerships share the same vested interests	Participation in planning and decision making
	Appropriate governance structures	Non-hierarchical relationship
	Common goals	Sharing of expertise
	Transparent lines of communication within and between partner agencies	Willingness to work together towards an agreed purpose
	Agreement about the objectives	Partnership
	Reciprocity	Interdependency
	Empathy	Highly connected network
		Low expectation of reciprocation

Reproduced with permission from [29].

In collaborations between a pharmaceutical company and representatives of other healthcare stakeholder groups (e.g. with HCPs, medical societies or institutions), a programme that addresses a well-defined educational gap is developed and implemented jointly, and roles and responsibilities of all partners are clearly defined. There are differences in how these collaborative frameworks are designed. Scientific committees are usually involved and work with pharmaceutical industry professionals from internal scientific/medical affairs departments to align on the objectives, appropriate governance and transparency requirements.

### Support to third-party-provided medical education (D)

Third-party-provided medical education programmes are supported *financially* by pharmaceutical companies through grants or sponsorships, and pharmaceutical companies have no involvement or influence in their development. The educational programmes are fully controlled by third-party medical education providers and these programmes follow the regulations, code of conduct and quality standards in medical education as set out by a recognised accrediting body. It is not mandatory for all educational programmes to be officially recognised as a contributing part of the continuous learning and development for HCPs; however, at the European level, accreditation is often a feature of third-party programmes and is provided by UEMS/EACCME or other international medical societies. At the country level, in accordance with national regulations, medical societies, universities or a variety of other accrediting bodies also have this authority.

## Discussion

It has been estimated that about half of all medical knowledge falls out of date within five years [31]. As the sequencing of treatments becomes increasingly complex and clinical practice evolves into individualised medicine, the necessity to update an HCP’s knowledge through CME-CPD is even greater. For the healthcare environment to operate at its maximum efficiency, deliver innovation, and result in improved patient outcomes, all stakeholders must constructively partner to optimise patient care, including initiatives directed to support high-quality medical education. The pharmaceutical industry is an integral part of this healthcare ecosystem, and through the different types of engagement pharmaceutical companies are playing a key role in innovation, education and collaboration. Indeed, the pharmaceutical industry has many experts who can contribute their expertise in these areas. This benefit has been acknowledged by key stakeholders such as the European Society of Cardiology, which states that “…it is essential that there is a strong collaboration between basic and clinical researchers from academic institutions on the one hand, with engineers and scientists from the research divisions of device and pharmaceutical companies on the other… promotion of advances by industry may accelerate their implementation in clinical practice” [6].

There has been significant progress made by the pharmaceutical industry over recent decades in creating an environment where positive contributions by pharmaceutical companies can thrive and malpractice can be eliminated. For example, to ensure ethical and transparent interactions in its activities and eliminate malpractice industry, authorities such as the European Federation of Pharmaceutical Industries and Associations (EFPIA) and other pharmaceutical associations at national levels were created, and defined external codes of conduct [20,32] including Compliance and Disclosure Codes from EFPIA and other local codes [15,16,32]. In addition to creating guidelines for a more fair and balanced approach, the Codes include transparency declarations such as fee-for-service to faculty and travel support for faculty or delegates – declarations not currently binding for any other type of medical educational provider. Further still, many pharmaceutical companies have proactively and purposefully created stricter controls with internal policies and procedures. Enforcement of these policies is ensured by mandatory training of employees, audit processes and governance provided by internal compliance teams. These examples, among others, have significantly contributed to readdressing a proper balance of integrity and ethics by ensuring an appropriate level of governance and transparency. It is largely due to these codes and processes that defining best practices for implementation and quality control has become an area of particular strength for the pharmaceutical industry in Europe.

Furthermore, in order to ensure appropriate focus on quality and a fair and balanced approach, over the past decade many pharmaceutical companies have established dedicated medical education departments, developed respective processes and appointed specialised functions that decide on the provision of grants and the development of educational programmes with collaborative involvement of external medical scientific experts. These efforts serve to ensure appropriate management of medical education and educational impact incorporating adult learning principles, as well as to minimise risk and perception of commercial bias.

In this context, two recently published, data-based, systematic reviews have analysed the relationship between commercial support and bias in CME [33,34]. The papers state that no data-based research could be identified supporting or refuting the hypothesis that commercial support produces bias in accredited CME. The reviews concluded that:

- Physicians reported the same level of perceived bias for programmes that were commercially supported and those free of commercial support [33,34].
- Overall, physicians perceived very low levels of commercial bias in post-programme course evaluations.
- Commercially supported CME can provide clinically accurate medical content [34].

In considering all the aspects above, these findings and developments do not support the current UEMS/EACCME regulations of restricting accredited programmes mainly to academia and medical associations. Indeed, conflict of interest and bias are not limited to the pharmaceutical industry or medical education [20,35]. The Oxford Dictionary defines bias as “a concentration on or interest in one particular area or subject” [36]. Every stakeholder in the healthcare ecosystem has their own concentration on or interest in one particular area or subject. Second, bias can also be an “inclination or prejudice for or against one person or group, especially in a way considered to be unfair” [36]. In essence, is it biased to restrict the role of the pharmaceutical industry with its skills, resources and expertise to just one financial provision? This inclination manages one bias by introducing another. This point appears to be frequently discussed in psychology journals. As stated in Baumeister’s paper *Bad is stronger than good*, when people make decisions about specific, impending events, they seem more motivated to avoid bad outcomes than to pursue good ones: “Indeed bad impressions and bad stereotypes are quicker to form and more resistant to disconfirmation than good ones” [37].

Mitigating one bias by introducing another can be counter-productive to achieving the programme objectives. Therefore, should not the focus be on eliminating inappropriate behaviours and conflicts of interest that are counter-productive to shared objectives, regardless of the provider, rather than on the existence of bias itself? The task of all stakeholders in today’s environment must be to ensure awareness of the ubiquitous risks and origin of bias and to develop structures, processes and control mechanisms to appropriately minimise risk while maximising all constructive contributions to improving patient care.

With this common goal in mind, would it not also be beneficial to harmonise the quality standards for medical education across the pharmaceutical industry *and beyond*? And to go one step further from the unilateral efforts being made by individual stakeholders and unite ourselves across the medical education environment? The quality criteria described in this paper were developed in order to initiate the discussion of such standards for the pharmaceutical industry; however, these criteria may have applications for all medical education providers to align on a cross-stakeholder vision on quality principles. There is much to be gained in uniting medical education standards in the various countries in Europe in a consultative setting led by respective professional or administrative stakeholder groups. When ultimately executing these standards it will be important for all providers to include appropriate internal training to ensure a high level of competence and compliance. It may also involve audits of any party providing medical education to ensure appropriate use of standards and possibly a formal qualification for medical education programme managers. In addition to contributing to the quality principles discussion, pharmaceutical companies have significant experience in harmonising local regulations and building international internal policies for company-wide execution within the existing codices, such as the EFPIA Code of Practice [15], which could be a role model for all stakeholders providing medical education [20]. The authors believe that the most reasonable way forward is for a multi-stakeholder group to collectively establish a robust quality process from planning to execution that can be applied in a consistent and transparent manner. This will need to be based on agreed criteria for quality, as well as organisation and transparency applicable to all providers of medical education.

## Conclusion

The ultimate objective of medical education is the optimal care of individual patients through improvement of public health and the effective use of available resources. There is, to date, a great deal of expertise, experience and knowledge in medical education management from various stakeholders, including the pharmaceutical industry, on which to capitalise. We must continue to break down silos among stakeholders in the provision of medical education. In Tom Stossel’s book *Pharmaphobia* he writes, “Manoeuvres that erect barriers to innovation and education are unacceptable. If we want biomedical innovation to succeed and medical education to be effective, we must remove barriers to cooperation, not impose them” [38]. In our opinion, recognising the pharmaceutical industry as an active collaborative partner in medical education will be beneficial for the healthcare ecosystem across the European region. To increase the overall quality of medical education in Europe, all stakeholders need to unite on clear criteria, with transparent roles and responsibilities and robust processes to effectively monitor and ensure quality and compliance. This will also help to harmonise medical education quality standards across Europe. Educational programmes that follow these criteria will become independent of the environment, the provider or the sponsor. We invite all stakeholders to come together and join an outcomes-oriented discussion on this new integrative model.
